# A qualitative study of a Sporting Memories program in South Australia: belonging, participation, and social connection

**DOI:** 10.3389/fpubh.2024.1424080

**Published:** 2024-07-30

**Authors:** Robert John Laidlaw, Richard McGrath, Saravana Kumar, Caroline Adams, Carolyn M. Murray

**Affiliations:** Allied Health and Human Performance, University of South Australia, Adelaide, SA, Australia

**Keywords:** aged, psychological wellbeing, quality of life, learning, loneliness, memory

## Abstract

**Background:**

Older people can experience health and social challenges such as loneliness, depression, and lack of social connectedness. There is need for programs and approaches that address the growing incidence of social isolation and loneliness for older people. One initiative that aims to address these challenges is the Sporting Memories program. This program was developed in the United Kingdom and licensed to South Australia in 2019. The program is currently delivered across six community locations.

**Methods:**

The aim of this study was to explore participants perspectives of the Sporting Memories program in South Australia. Underpinned by qualitative research, three focus groups were conducted, led by an experienced interviewer. Focus groups occurred at three of the six locations, including a day respite center, assisted living center and a government community center. The data were analyzed thematically by the research team.

**Results:**

There were 16 participants over 65 years old, including four women and 12 men. Three key themes were developed: “free to talk about anything,” “not feeling left out” and “a chance to share and learn.” Collectively, participants reflected on how they built social connections, felt safe and included and learnt more about each other.

**Conclusion:**

The Sporting Memories program provides a group program for older people to come together and develop new friendships. The use of sports as a means of reminiscence was considered relatable for the participants who reported social benefits and plans to keep attending. They valued learning through the program which was enhanced by having a facilitator who was knowledgeable about sport.

## 1 Introduction

Over the course of the past 30 years, there has been a remarkable increase in life expectancy across much of the developed world (1). While increases in lifespan and decrease in mortality is a positive development for humanity (2), it comes at a price. Australia, like much of the western world, confronts a growing aging population (3). The population of people aged 65 or older in Australia has increased from 4.6% in 1922 to 16.2% in 2021 with growth to continue, with over 20% expected by 2066 (3). Between 2000 and 2050 alone, the global share of people 80 and older is estimated to be almost 5% (4). Catering to the aging population requires adequate resourcing of the aged care sector and workforce to manage complex health and social issues. However, declining fertility and shrinking size of the working age population compound these challenges (2).

In addition to the well-recognized physical impacts of aging (1), there are also socio-cultural challenges associated with aging (2). For example, as people get older, they may experience cognitive changes, bereavement (such as loss of a life partner) and drop in income. These experiences can all contribute to social isolation, loneliness, loss of independence and increased psychological distress (5). Research indicates that with increasing age, older people experience higher levels of psychological distress (6). A review of the literature identified that social isolation and loneliness can substantially increase the risk of dementia, coronary artery disease and all-cause mortality (7).

Different approaches have been trialed to address the negative health impacts of social isolation and loneliness in older people including at the community level (such as the Campaign to End Loneliness in United Kingdom),^1^ directly (such as social prescribing, support groups, cognitive behavioral therapy, mindfulness and pharmaceutical interventions) and indirectly (such as participation in exercises, and gym memberships to promote broader social engagement) (8).

Another direct approach to counter social isolation and loneliness is through reminiscence programs. Reminiscence is verbalizing past events as remembered by the narrator, which, can encourage the wellbeing of participants through sharing of stories. Reminiscence is based on past experiences, and although often reliant on an individual’s memories, can be a catalyst for connecting with others who have had similar experiences (9–11). Reminiscence is described as memories, recollections, reflections, remembrances, anecdotes, or memoirs, with its application for health benefits being of interest for a growing aging population (12). Types of reminiscence can include integrative (promoting self-worth through reconciling past events), instrumental (recalling past coping strategies that lead to resilient responses), transmissive (passing on wisdom and legacy), narrative (describing or telling stories), escapist (preferring the past to the present), and obsessive (rumination over past regrets or guilt) (13). Of these, the last two could be regarded as unhelpful processes for healthy aging, with some evidence that the first four can by psychosocially positive, particularly if conducted in group settings (7, 11). Reminiscence can incorporate “tools” such as art, music, poetry, objects, photographs, and activities (12).

Reminiscence can draw on a range of past experiences, including sport. Sporting Memories programs use reminiscence about individual’s past sports experiences and were originally developed in the United Kingdom and registered under the Sporting Memories Foundation.^2^ The aim of the program was to bring together older adults to talk about and remember sporting experiences, while providing social and physical activities to provide an atmosphere of fun and friendship, thereby improving wellbeing (10, 14). Sporting Memories programs attract people with an interest in sport, who may not engage in other mediums such as music or art. The programs have been developed to support the wellbeing of people who may be living with cognitive changes, loneliness and/or depression (10). During sporting reminiscence, various stimuli or props are used, including video, photos, newspaper articles, sporting memorabilia and personal stories. The sporting memories programs can also incorporate sensory stimulation such as sights, sounds, tastes, smells, through exposure to memorabilia, music and food from the era where a sporting memory is being recalled (10).

### 1.1 The context

In 2019 SportsUnited was granted a license to run Sporting Memories programs in Australia. In 2024, there were at least six locations operating regularly in South Australia, with programs organized weekly, fortnightly, or monthly. Sporting Memories sessions are delivered across a range of locations, including local government community centers, aged care supported accommodation and aged care community centers. Sessions are devised to support the mental and physical wellbeing of people aged over 50, with a focus on using memories from sporting activities and involvement to assist with achieving the program goals. Participants in Sporting Memories programs can include people who are identified as socially isolated and/or lonely, affected by depression and/or anxiety, as well as those who may have mild cognitive changes. To prompt group discussions during Sporting Memories sessions a range of activities are conducted including games, quizzes, memorabilia, guest speakers and trips to sporting venues.

A systematic review of community-based reminiscence programs was conducted by the authors in 2023 which identified 27 studies (17 quantitative and 10 qualitative) (12). This review demonstrated positive findings from reminiscence programs with a reduction in depression, anxiety, and loneliness reported as well as improvements in quality of life and mastery. These findings were supported and broadened by qualitative findings with three key themes outlining program processes, necessary program ingredients, and program benefits. Key program processes included having a program manual, a well prepared facilitator and programs situated in the community rather than in clinical settings. Key ingredients included defining the group purpose, having inclusive activities that had a consistent focus and left scope for playfulness, humor and some physical activity. Key benefits included an improved sense of self-worth and development of relationships with others (12). The findings of this review were supported by previous literature (15, 16) and formed the basis for the current primary research.

### 1.2 Study aim

As there has been limited research exploring participant perspectives of the Sporting Memories program both in the UK and in Australia (15), the aim of this study was to explore older people’s perspectives about the Sporting Memories program offered in metropolitan Adelaide, South Australia.

## 2 Methods

### 2.1 Study design

As the purpose of the research was to gather perspectives of Sporting Memories attendees, a qualitative descriptive methodology was used (17, 18). This approach sits within the interpretive paradigm and is well suited to exploring perspectives about a community program because it enables gathering of information rich descriptive experiences from participants (17, 18). Focus groups were the chosen method of data collection because they enable data collection within the natural contexts of the group environment and discussion among the group members stimulates thinking and contribution (19).

### 2.2 Ethics statement

This study received ethics approval from the Human Research Ethics Committee of the University of South Australia (protocol number: 205846).

### 2.3 Positionality statement

The first author (RL) has an active interest in sporting history and has a lifetime of involvement in sport, as a participant and official. RL assists with delivery of the Sporting Memories programs in South Australia, while RM and CA are involved with SportsUnited and support implementation of the Sporting Memories program. The other authors (CM and SK) have no connection with Sporting Memories or SportsUnited. All authors have an interest in the value of reminiscence programs and the need to support older people to sustain social connections and mental health.

### 2.4 Sampling and recruitment

Three sites were purposively sampled for inclusion in the study to ensure diversity in the data set. These sites included a community day respite service, an assisted living community and a local government community group. The site managers of three sites were approached by the SportsUnited organization to give them information about the research and invite them to participate. All the sites who were invited agreed to participate.

Within each of these sites, participants for the focus groups were conveniently sampled based on being regular participants in the Sporting Memories program (20). People voluntarily attend the Sporting Memories program and are usually over the age of 65. Some have cognitive changes, but the program is not suitable for those with advanced dementia. The manager for each site distributed participant information sheets and consent forms to those members who regularly attend the Sporting Memories group. The manager at the participating site arranged the consent process and where they believed it was necessary, they sought consent from the person’s next of kin. Communication from SportsUnited with Sporting Memories participants occurred via the site manager is usual.

### 2.5 Data collection

Questions for the focus group were conducted based on the findings from the prior systematic review that was conducted by the research team (12). Questions were designed to be open-ended and exploratory to elicit discussion and avoid leading the group. The details of the questions asked are provided in Table 1. Where required, follow up and prompting questions were asked.

**Table 1:** Focus group guide.

Questions asked
1. What is your understanding of the Sporting Memories program? 2. How did you feel about being involved with the group? 3. What aspects of the program were satisfying for you? 4. hat were the best experiences of being a participant? 5. hat suggestions do you have for improving the program? 6. How would you describe this program to a friend?

Given some members of the research team were involved in the delivery and implementation of the Sporting Memories program at the sites, a member of the research team with no prior involvement (CM) conducted the focus groups. CM has extensive experience in qualitative research and collection of data through interviews and focus groups. The participants received a shortened Sporting Memories program delivered by RL and then those who had consented transitioned into the focus group. To encourage people to speak freely, the focus group occurred in a private room with no other members of the research team present other than CM. The focus groups were an average duration of 28 min (range 21–41 min). The group members already knew each other as they all participate in the Sporting Memories program, meaning that positive group dynamics and familiarity were already established. There were no incentives or rewards provided for focus group participants.

### 2.6 Data analysis

The reflexive thematic analysis approach of Braun and Clarke was followed (21). This approach guides management of data through a process of coding, synthesis and thematization and ensures authors are reflexive during data interpretation. Firstly, the focus groups were audio-recorded and manually transcribed verbatim by RL. At this point RL assigned each participant a pseudonym to protect participant identity. The accuracy of each transcript was checked against the recording by another member of the research team (RM, CA, and CM). Secondly, line by line assignment of codes was conducted within each transcript independently by two members of the research team (RM, CA, CM). The research team then came together to discuss patterns seen across the codes and develop five preliminary themes. A second round of data analysis was conducted by the team to confirm initial theme identification. These themes were double checked against the transcripts (raw data) to ensure their dependability (20). The research team then met again to discuss the definitions and naming of themes thus reducing them to three. Quotes were selected as supporting evidence of themes. Supplementary File S1 provides an overview of the rounds of analysis and the process of refining and synthesizing data to final themes.

### 2.7 Rigor

Having an independent researcher facilitating the focus groups supported participant honesty thus improving the quality of the data. Bias was also minimized in interpretation of the data through the reflexive team approach to analysis, openly declaring biases and assumptions up front and regularly throughout the process and keeping reflexive memos of reasoning behind decisions made. An audit trail was kept of analytic decisions by saving different versions of documents at each stage of the analysis (to enable backtracking) (18). To stay grounded in the data, the developing themes were regularly checked against transcripts and participant quotes were used to support the themes. These quotes use the pseudonym to identify the participant and the focus group (FG) they participated in.

## 3 Results

### 3.1 Participant characteristics

There were 16 participants, all over the age of 65. Focus group one occurred in a day respite center, focus group two in an assisted living center and focus group three was in a local government community center. Focus groups one and three consisted of only men with focus group two having four women and three men. Any participants showing signs of cognitive changes participated with extra verbal prompts and time to process questions. One participant had expressive aphasia and one did not have English as a first language; but both participated actively with the facilitator, who noted relevant non-verbal language to support verbal contributions. To protect participant anonymity, these details are not linked with pseudonyms and focus group number.

### 3.2 Descriptive themes

Three key themes were identified to describe the experiences of those participating in the Sporting Memories programs. These themes included “free to talk about anything,” “not feeling left out,” and “a chance to share and learn.” Collectively these themes describe how the atmosphere within the groups enabled the participants to speak freely and candidly. The consistent structure and culture within the groups meant they were regarded as inclusive and gave an opportunity to “lean in” and learn more from the other group members, the facilitators and themselves. While there was both males and females in one focus group, data analysis did not reveal any differentiation of findings based on sex.

#### 3.2.1 Free to talk about anything

This theme explains how the participants valued being able to speak freely with no judgment: *“you can put in your two bobs worth, and no one laughs”* (Vic, FG 3). Exchanges were often tinged with humor though there was no malicious intent. As Ken (FG 3) pointed out, “*I like the roasting part where, you know, somebody might come up and have a go and all that and I can have a go back.”* This humor was demonstrated during the below interaction between four male participants in focus group 3:

Ken – *“I can remember Jack when he had brown hair”* [laughter].

Jack – *“Yeah, there was a time.”*

Vic – *“You need to remember when Ken did have hair too.”*

Ken – “*Yes.”*

Vic – *“Did have ….”*

Ken – *“Yes, I was … just changing from a nappy.”*

Vic – “*ohh I get it. Yeah.”* [laughter].

Toby *“The banter you have got going on here now is typical … It’s quite pleasing. You feel quite proud of the group.”* (FG 3).

The focus on sports was attractive to participants with Darren (FG 3) and Tom (FG 1) identifying this as the initial reason for attending sessions:

*“I enjoy coming here, you know. Talking to people about sports, talking to people about what I’ve done in sports.”* (Darren FG 3).

*“Talk about everything, footy, cricket, netball and everything. What everyone wants to talk about”* (Tom FG 1).

Paige (FG 2) described how she did not like to brag but she enjoyed talking about her achievements and showing her sporting trophies and found it was well received by the group.

*“I took a couple of trophies and things, and Brenda took her hockey trophy, and that was a discussion ….”* (Paige, FG 2).

Several participants expanded the conversation away from sports to discuss family and health issues and provide feedback about other activities outside of the Sporting Memories program.

*“… we were able to touch on cancer … and it’s good just to open up a little bit.”* (Vic, FG 3).

*“… a small get together, basically a sporting group that also speaks of other things. Personal things, experiences. And I’d also say it does not get too serious.”* (Jack, FG 3).

#### 3.2.2 Not feeling left out

This theme explains how the participants described the program as inclusive. They attributed the inclusivity to the encouragement provided by the facilitator as well as the relaxed and *“intimate”* (Toby, FG 3) atmosphere. The size of the group was discussed as needing to be 6–8 people to keep it *“informal”* (Darren, FG 3). The *“laidback attitude”* of the facilitator *“encourages all to have our say” (Rick, FG 2)*. Participants reported that during the group program, the facilitator would offer hints, ask questions, give space, and guide the conversation to ensure no-one was left out.

*“He* [facilitator] *gives you a couple of hints to put you on the right track”* (Ken, FG 3).

*“He’s [facilitator] able to extract things out of people … can ask a question quite delicately … but it gets a response.”* (Don, FG 2).

Vic (FG 3) was adamant that people be allowed to sit back and contribute on their terms: *“we are gonna (sic) talk when we wanna (sic) talk, you know”* (Vic, FG3). Rick (FG 2) agreed that he did not say much during the group, but he got *“a lot of pleasure out of coming and just listening to what other people have to say.”* Vic (FG 3) stated that *“we do not want structure”* and the reasons for this appeared to center on keeping inclusivity and pressure off people.

*“I just want to sit back and enjoy the company without pressure”* (Vic, FG 3).

*“Once you start bringing your formal structure into this sort of area, it ceases to have the same impact.”* (Toby, FG 3).

#### 3.2.3 A chance to share and learn

This theme explains how the reminiscence function of the program meant that participants learned more about others in the group. This sharing of memories, achievements and personal stories was a catalyst for people to get to know each other better which consolidated friendships.

*“I did not know much about Jack until we sort of started” (Vic, FG 3).*

*“People do not realise, that I’m still learning the amount of sport that Paul has been involved in.” (Don, FG 2).*

Participants acknowledged that they enjoyed how knowledgeable some other group members were. Ken (FG 3) was surprised *“the knowledge that’s going around … what comes out different guys, they have got different things that have happened to them.”* Paul (FG 2) concurred; “*I do not mind sharing … (and) I’m interested in what other people have done.”* Similarly, Leanne (FG 2) enjoyed *“… gathering information on what other people have done.”* Vic (FG 3) found other people’s stories *“… most times, damn interesting.”*

The other mechanism for learning was the role of the facilitator who was described as *“knowledgeable*” (Ken, FG 3) and *“like a good school teacher … it’s all part of education and all part of sport, which is interesting”* (Don, FG 2). Leanne (FG 2) described the program as *“informative*,” and the perceived educational purpose of the program came up multiple times:

*“…we all learn something every time” (Jack, FG 3).*

*“… oh it’s pretty good, real good, and I learn a lot”* (Tom, FG 1).

The process of reminiscence was valued by group members who described reflecting and remembering in a setting where they did not feel pressure to give answers if they were not available to them:

*“…helped me a lot in, we’ll say … stuff that I cannot remember, but bringing it out …” (Ken, FG 3).*

*“… for me, it’s to get in touch, a little bit, with your past with sporting, but not to the point where it overwhelms you.” (Vic, FG 3).*

## 4 Discussion

The aim of this study was to explore the experiences of participants in the Sporting Memories program. Three focus groups from three diverse locations were conducted, involving 16 participants from the Sporting Memories program. Collectively, three themes were identified namely “freedom to talk about anything,” “not feeling left out” and “a chance to share and learn.” Findings from this study provide insight into the wide-ranging positive impacts of reminiscence programs, regardless of sex. In addition to the reported health and social benefits, older people also valued the opportunity to learn about, and from, each other. A knowledgeable facilitator was seen to be critical in bringing the group together and promoting inclusivity. Collectively, participants described the Sporting Memories program as a chance to build social connections.

Building social connections through developing new relationships and meeting new people has been found in other studies exploring reminiscence programs (22–27). These studies explore reminiscence programs located in community facilities and aged care environments across several regions (including the United States, United Kingdom, and Asia). They had different foci such as creative activities (22), multimedia (23), computer mediated communication (24), museum visits (26) and music (28). The focus of sports used in the Sporting Memories program contributes positively to the suite of options available for delivering programs that assist older people, to develop social connections, decrease depression and loneliness and increase quality of life among older people (12). The existing literature related to Sporting Memories has stated that men are more likely to participate in their programs (10, 14, 15). However, to date there is no research evidence to suggest that there are any differences in program outcomes depending on the gender of participants.

The roles and skills and facilitators in delivering reminiscence programs has been identified as key to engagement and participation (12) and was also identified by participants in this research. Trained facilitators can create safe environments (23, 29), and need to be prepared to adapt and draw on different approaches according to the group dynamics (30). Findings from this study indicate an inclusive environment was created by the facilitator, through providing participants a chance to share their stories, and the freedom to talk about anything. The inclusivity seemed to relate to the informal structure delivered through the design of the Sporting Memories program but also from the way it was delivered. The Sporting Memories sessions use “simple reminiscence,” which is described as spontaneous reminiscence without structure that aims to increase social wellbeing of older people (31). Having said that, the Sporting Memories program is not entirely unstructured because the facilitator arrives with a plan (structure), props and stimuli; ready to be flexible based on the group dynamic on the day. Another ingredient for inclusivity identified by focus group participants were group sizes between six to eight people which is also reported as an ideal group size by Syed Elias et al. (31).

Learning from other participants and the facilitator as an outcome of community-based reminiscence programs appears not to have been identified in previous studies. Often previous studies have focused on health and social outcomes (12). Learning for older people has been discussed within frameworks such as active aging (32) and lifelong learning (33) with research finding that for some older people providing opportunities to actively engage with learning activities on topics that they have some interest in stimulates cognitive functioning and social engagement (33, 34). Learning for older people is important (35) because it fosters an active and enquiring mind, broadens horizons as well as promoting social interaction through staying connected to society (35). As such, providing older people the opportunity to learn as an element of the Sporting Memories program appears to be valued by participants meaning they continue to attend and consolidate their social connections and friendships.

## 5 Strengths and limitations

A strength of this study was the diversity across settings for the focus groups contributing to richness in the data collected. Having an interview guide that was informed by the literature also contributed to depth of data collected. Data were collected by an independent, experienced qualitative researcher who was able to note non-verbal communications, ask probing questions where needed and encourage participation. As with any research, there are limitations to consider. As this research involved participants from Sporting Memories reminiscence programs, with a small sample size, the findings may not transfer to other reminiscence programs. Having more male than female participants in the focus groups in the sample related to the people at the sites where the focus groups occurred rather than the Sporting Memories program being preferred by men. Data about ethnicity were not collected about the participants. Future research could aim for a balance of gender and reporting information about ethnic backgrounds of participants. The nature of the focus groups meant some participants may have been influenced by the contributions of others.

## 6 Conclusion

Exploring participant perspectives of three Sporting Memories programs in South Australia has provided important insights. The program is an innovative community-based initiative that can be tailored to participant interests, experiences with and memories of sport. While Sporting Memories appears to contribute to improved health and social connections, the positive effects extend to learning. Reminiscing about a universally accessible activity, such as sports, can foster opportunities for older individuals to connect and learn about, and from, each other. These experiences were enhanced by a knowledgeable facilitator promoting positive group dynamics through inclusivity and flexible structure. Given these findings, it is recommended that facilitators of reminiscence programs be suitably skilled and trained to promote optimal experiences for participants. Similar research conducted with other community reminiscence programs, as well as longitudinal and quantitative research that compares outcomes from sports reminiscence with other types of reminiscence could strengthen the evidence base.

## Data availability statement

Requests to access the dataset should be directed to the corresponding author. Any release of data would be subject to approval from the Human Research Ethics Committee that approved the project.

## Ethics statement

The studies involving humans were approved by the University of South Australia, Human Research Ethics Committee. The studies were conducted in accordance with the local legislation and institutional requirements. The participants, or their next of kin where necessary, provided written informed consent to participate in this study.

## Author contributions

RL: Conceptualization, Data curation, Formal analysis, Methodology, Project administration, Writing – review & editing, Investigation, Writing – original draft. RM: Conceptualization, Data curation, Formal analysis, Methodology, Project administration, Writing – review & editing, Supervision. SK: Conceptualization, Data curation, Formal analysis, Methodology, Supervision, Writing – review & editing. CA: Conceptualization, Formal analysis, Methodology, Supervision, Writing – review & editing, Data curation. CM: Conceptualization, Data curation, Formal analysis, Methodology, Supervision, Writing – review & editing, Investigation, Project administration.

## Footnotes

^1^https://www.campaigntoendloneliness.org/

^2^https://www.sportingmemories.uk/
