# Supplementary material for: A qualitative study of a Sporting Memories program in South Australia: belonging, participation, and social connection
Source: Front Public Health. 2024 Jul 30;12:1424080. doi: 10.3389/fpubh.2024.1424080 (PMC11319177; doi:10.3389/fpubh.2024.1424080)
Supplement: Supplementary file 1 [file Table_1.DOCX]

Supplementary File 1: development of themes

| **Round 1 of analysis** | **Round 2 of analysis** | **Final themes** |
| --- | --- | --- |
| Free to talk   - More power and voice - Relaxed - No pressure - Banter | Freedom to talk about anything   - Comfort and feeling safe - Opening up - Talk about other topics - Banter | Free to talk about anything |
| Approach of facilitator   - Inclusive - Encourages - Gives prompts - Comes back to everyone | Do not feel left out   - Inclusive - Open minded - Intimacy - Encourage reticent participants   Hear what people have done   - Friendship - Storytelling - Social contacts | Not feeling left out |
| Learning something   - Time to discuss - Knowledgeable facilitator | Learn a lot   - Reminiscence - Rekindling memories - Curiosity | A chance to share and learn |
| Being with others   - Laid back - Interactions encouraged - Sparking memories | Rapport and connections   - Comradeship - Keep group small - Informal structure - Trust/ laid back |  |
